# Adipose-Derived Stem Cells in Radiotherapy Injury: A New Frontier

**DOI:** 10.3389/fsurg.2015.00001

**Published:** 2015-01-28

**Authors:** Lipi Shukla, Wayne A. Morrison, Ramin Shayan

**Affiliations:** ^1^Regenerative Surgery Group, O’Brien Institute, Fitzroy, VIC, Australia; ^2^Department of Plastic Surgery, St. Vincent’s Hospital, Fitzroy, VIC, Australia; ^3^Regenerative Surgery Group, Australian Catholic University and O’Brien Institute Tissue Engineering Centre (AORTEC), Fitzroy, VIC, Australia; ^4^Department of Surgery, University of Melbourne, Melbourne, VIC, Australia

**Keywords:** radiotherapy, adipose-derived stem cells, soft-tissue injury, autologous fat grafting, cancer, radiation, reconstruction

## Abstract

Radiotherapy is increasingly used to treat numerous human malignancies. In addition to the beneficial anti-cancer effects, there are a series of undesirable effects on normal host tissues surrounding the target tumor. While the early effects of radiotherapy (desquamation, erythema, and hair loss) typically resolve, the chronic effects persist as unpredictable and often troublesome sequelae of cancer treatment, long after oncological treatment has been completed. Plastic surgeons are often called upon to treat the problems subsequently arising in irradiated tissues, such as recurrent infection, impaired healing, fibrosis, contracture, and/or lymphedema. Recently, it was anecdotally noted – then validated in more robust animal and human studies – that fat grafting can ameliorate some of these chronic tissue effects. Despite the widespread usage of fat grafting, the mechanism of its action remains poorly understood. This review provides an overview of the current understanding of: (i) mechanisms of chronic radiation injury and its clinical manifestations; (ii) biological properties of fat grafts and their key constituent, adipose-derived stem cells (ADSCs); and (iii) the role of ADSCs in radiotherapy-induced soft-tissue injury.

## Mechanisms of Radiotherapy-Induced Soft-Tissue Injury

**Figure 1:**
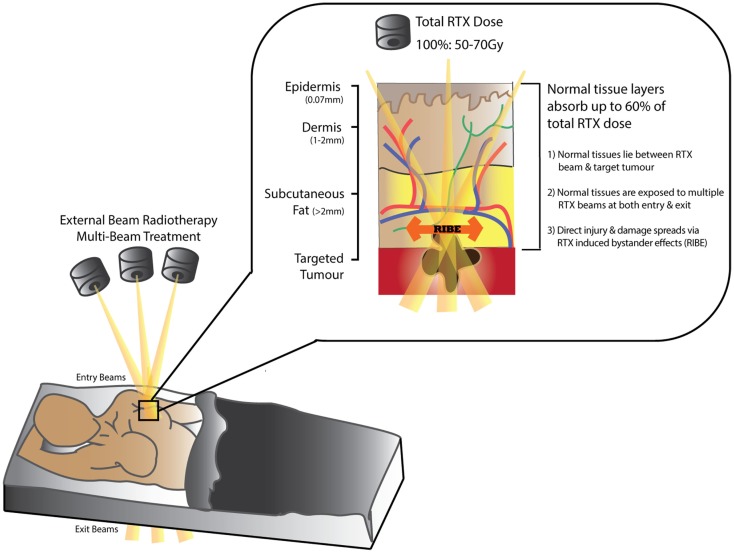
**Schematic diagram demonstrating tissue absorption of external beam radiation administered in radiotherapy (RTX)**. The RTX beam interacts with living tissues resulting in electron excitation, release of energy, and damage to both tumor and normal tissue cells. Normal tissues can absorb up to 60% of the total RTX dose targeting the tumor. Measurements in (mm) represent distance from skin surface.

Over 50% of patients diagnosed with cancer (excluding non-melanoma skin cancer) (1, 2) require Radiotherapy (RTX) for curative or palliative treatment (3–8). While RTX is classified as a “non-invasive” treatment modality, dose-delivery is limited by the capacity of surrounding normal tissues to tolerate radiation exposure (Figure 1). The acute/early side-effects (within 10–14 days) are most obvious in rapidly proliferating cells such as epithelial surfaces (epidermis and intestinal or respiratory mucosa) and bone marrow; and include desquamation, swelling, erythema, and pain (2, 4, 9). These sequelae are followed by more unpredictable and progressively worsening late-onset chronic side-effects (months/years after treatment) (10–15). Recent advances in public awareness, early detection, and adjuvant cancer therapies have led to significant improvements in cancer survival rates (30% in recent decades) (2, 5, 16, 17). Consequently, more patients are living longer with a wider range of chronic RTX-related morbidities that impair their quality of life and increases their burden of disease (18); as well as leading to potentially life-threatening complications. Furthermore, in the case of cancer recurrence following RTX, treatment options become higher-risk and reconstructive surgical options more limited (19).

While acute manifestations of RTX are due to loss of functional cells through either apoptosis or cell death (20, 21), late RTX effects are less likely to arise due to these mechanisms (22). Instead, the initial sub-lethal indirect tissue damage results in an evolving disruption of key cellular repair mechanisms (23). Stewart et al. suggested that altered molecular signaling and formation of reactive oxygen species (ROS) cause single-stranded DNA breaks that repair incompletely, activating premature senescence, or accelerated differentiation pathways (21). According to Haubner et al. and others, these changes result in delayed RTX side-effects by further eliciting persistent localized cellular dysfunction, well after the early tissue reactions have subsided (6, 9, 10, 21, 24–31). Stem cells within the injured area further recruit myofibroblast-like cells, which in turn contribute to chronic fibrosis (32, 33).

Meanwhile, Stone et al. hypothesized that in addition to losing reproductive capacity altogether, irradiated cells harbor sub-lethal injuries that perpetuate cell dysfunction through ongoing transcription of damaged DNA (9, 34, 35). Therefore, the pathogenesis of RTX-injury is now considered a continuum of events that propagates damage to surrounding normal tissues, rather than a simple acute injury that creates an area of scarring (21, 36). Emerging work further suggests that an organized active biological release of inflammatory chemokines and cytokines may also establish a chronic inflammatory state in irradiated tissues (1–8, 10, 21, 24). Additionally, irradiated tissues also fail to regenerate normally when subsequently injured i.e., RTX-injury is a disease in and of itself.

In terms of the specific molecular signals implicated in the pathogenesis of RTX-injury, up-regulation of the TGFβ signaling is a mechanism common to numerous conditions of pathological fibrosis (2–4, 9), including fibrosis following cancer treatments such as radiotherapy or chemotherapy (5, 7, 10–13). Brush et al. suggest that the impairment of normal healing results in compensatory hyper-activation of fibrotic pathways, in order to maintain tissue structure and integrity (10, 14, 15). Work by Lee et al. demonstrated persistent TGFβ-1 over-expression in irradiated tissues, even after 6 months (2, 14, 16); alterations that may in turn influence the function of fibroblasts, endothelial cells, lymphocytes, macrophages, and platelets (5, 7, 17). Tibbs et al. characterized the key cellular functions of TGFβ, including initiation of tissue matrix production and stimulation of chemotactic migration of fibroblasts and monocytes (12, 18). In contrast, Randall et al. showed oscillating TGFβ-1 expression – decreased in the first 3 h after RTX (normalizing by 2–7 days), then steadily increasing to up to 200% above normal levels more chronically (16, 19). Grose and Werner verified a role for TGFβ in RTX-induced fibrosis and investigated the modulation of downstream mediators such as Smad-3 (17, 20, 21). They demonstrated accelerated re-epithelialization and decreased inflammation in Smad-3^−/−^ mice compared with control animals (17, 22). Despite this evidence, however, attributing specific cellular effects of RTX-induced fibrosis to such a broad regulator of fibrosis as TGFβ has its limitations. The TGFβ super-family has multiple effects on numerous tissues and therefore therapeutic approaches that target this molecule may have insufficient specificity to ameliorate RTX damage, without jeopardizing other biological processes to which fibrosis is integral.

The focus of clinical and scientific research investigating RTX has, therefore, begun to shift from the initial insult to the modulation of subsequent processes such as inflammation (37, 38) and repair/remodeling (6, 9, 20–26), in order to reduce harmful sequelae of RTX-induced soft-tissue injury. Impaired regeneration of irradiated tissues may also arise through a lack of available stem cells to mediate the repair process (see below). Finally, microvascular damage and lymphedema are also emerging as key features of chronic radiation injury; and it is in the light of shifting paradigms in our understanding of the field that we present a review of experimental and clinical adipose-derived stem cell (ADSC)-based approaches to RTX-induced soft-tissue injury to date. NB: Although, RTX dosage and delivery regimes are related to potential morbidity, they are beyond the scope of this review.

## Biological Properties of Fat Graft and ADSCs

**Figure 2:**
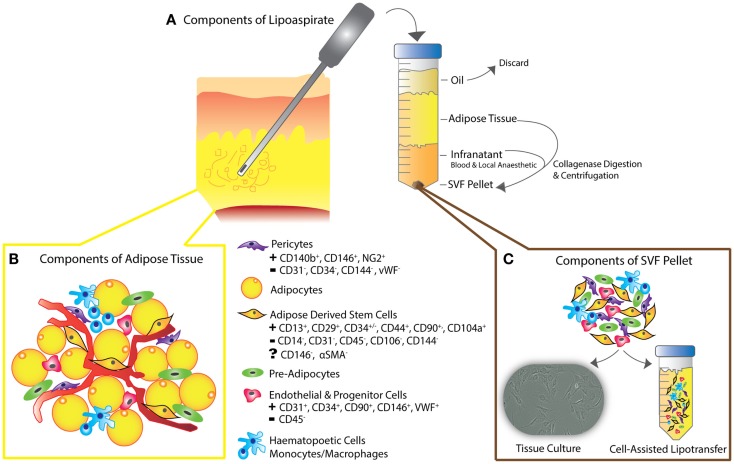
**(A)** Schematic diagram depicting liposuction procedure – lipoaspiration of subcutaneous fat is performed, as previously described (30), followed by separation into layers of oil (discarded), aspirated adipose tissue, and infranatant (composed of blood, plasma, and local anesthetic). **(B)** The components of adipose tissue and the key constituents of the SVF pellet are all present in en-bloc *in vivo* adipose tissue as shown. Following collagenase digestion, incubation in control medium and centrifugation, the residual pellet is the so-called stromal vascular fraction (SVF). **(C)** SVF can be plated for tissue culture or added to unprocessed lipoaspirate as in the process of “cell-assisted lipotransfer” (43). The key surface markers of ADSCs, pericytes, endothelial, and progenitor cells are shown, demonstrating the unique surface antigen profile of each cell type that allows their differentiation from ADSCs (smooth muscle cells and fibroblasts not shown).

Adipose tissue is heterogeneously distributed around the body and variable between individuals. Fat is mainly composed of lobules of mature adipocytes, and has mechanical and esthetic functions as well as roles in metabolism – a highly specialized type of connective tissue responsible for insulation, protection, and energy regulation (21, 25, 27). The bulk of the non-adipocyte component, the cells within the stromal vascular fraction (SVF) are from mesodermal or mesenchymal origin and include pre-adipocytes, fibroblasts, endothelial cells, vascular smooth muscle cells, immune cells, and ADSCs (Figure 2) (27–31, 39–42).

Plastic surgeons use fat in vascularized tissue flaps, non-vascularized composite grafts, or stand-alone grafts in fat transfer (28, 30, 32). The relative abundance of adipose tissue in most patients and ease of obtaining fat by lipoaspiration/liposuction with minimal donor morbidity has expanded the range of clinical indications for fat grafting; such as correcting cosmetic or contour defects, contractures, and lymphedema (30, 33, 44, 45).

Initially in clinical observation (9, 30, 36), then in animal models (15, 34, 46); fat grafting was reported to improve the characteristics of overlying skin and soft-tissue in RTX-injury (6, 30, 35, 46, 47). Subsequent clinical analysis verified softening of wrinkles or fibrotic tissue and resolution of pigment changes (21, 36, 39, 46, 48). Clinical reports suggested that fat grafting may also reduce peri-prosthetic capsule contracture, vocal cord damage, and chronic ulceration; and that it may rejuvenate aging skin (5, 27, 28, 36, 39, 41, 46–52). These clinical benefits were attributed to the regenerative properties of undifferentiated multi-potent ADSCs within the SVF of lipoaspirate (36, 53). ADSCs are thought to play a supportive role in adipogenesis and angiogenesis, while also modulating inflammation and immunity (30, 54–56). Therefore, a role for ADSCs/fat graft ameliorating RTX-injury would be of interest to those working in tissue engineering, regenerative medicine, and clinical plastic surgery.

However, despite promising clinical potential, a detailed understanding of the putative molecular mechanisms for ADSC-mediated reversal of RTX-injury remains elusive (36, 44, 57). Additionally, concerns have been raised that fat grafting following cancer treatment may enhance tumorigenesis in a former cancer bed (58–60). If fat grafting is to become a useful and validated clinical tool, these issues must be addressed. A thorough understanding of the molecular interactions and the functional and sub-cellular alterations caused by RTX-injury to ADSCs themselves is also needed. Without such insights, guidelines pertaining to the safety of fat grafting in these contexts cannot be developed (43, 60, 61).

## Enhancing Fat Graft Take Using ADSCs

Due to the clinical origins of the field, the majority of mechanistic information regarding ADSC-mediated cellular effects has been derived from research investigating the enhancement of fat grafts. Therefore, in understanding what pathways may become activated in ADSC-mediated reversal of RTX soft-tissue injury, it is critical to first review this more well-established body of data.

A tissue graft is defined as autologous tissue transferred to a distant site, without its original blood supply. A fat graft therefore, must acquire a blood supply and nutrients from the tissue bed into which it is introduced, with early re-vascularization to prevent graft necrosis that leads to volume depletion (50, 62, 63). Unfortunately, fat grafts may resorb up to 70–100% of the initial injected tissue volume (30, 64); a result attributed to poor graft neo-vascularization, apoptosis, and/or chronic fat necrosis (39–43, 60, 63–65). While the many technical modifications to enhance fat graft take are beyond the scope of this review, ADSCs have emerged as a key focus of graft enhancement, and more recently as a critical component in reversing soft-tissue injury (66). ADSCs, first isolated by Zuk et al. over a decade ago (28, 67), were postulated by Eto et al. to be more robust than mature adipocytes in resisting mechanical trauma during fat transfer (30, 42, 44, 68), and to have lower metabolic demands (27, 28, 39, 48, 49, 62, 69–72). Others demonstrated improved graft survival through increased angiogenesis, incorporating either imported endothelial progenitors or ADSCs into blood vessels (52, 53, 72). In contrast, Butala et al. suggested that introduced ADSCs may recruit further stem cells, particularly from bone marrow (30, 53, 55, 72). To enhance the relative ADSC abundance within fat grafts (44, 58, 72, 73), Yoshimura et al. proposed “cell-assisted lipotransfer enrichment” (Figure 2), by supplementing lipoaspirate with additional SVF (23, 43, 60, 62, 64, 74, 75). The SVF [comprised 10% ADSCs (45, 46, 65, 67)], is obtained from a component of lipoaspirate, surplus to the volume anticipated to be required to fill a known defect (30). This surplus lipoaspirate is separated into components by centrifugation (Figure 2). Following collagenase digestion, further spinning produces a pellet, referred to as SVF. Finally, the SVF is re-introduced to the remaining lipoaspirate, in preparation for injecting the ADSC-enriched fat graft (28, 39, 42, 62, 68, 76–78). Later, Piccinno et al. explored graft enrichment using *in vitro* purified and expanded ADSC populations (69, 79), while Lu et al. and Shoshani et al. performed co-injection of pro-angiogenic factors IL-8 and VEGF-A (70, 71, 73). These studies collectively suggested that such enrichment may further increase graft viability, neo-vascularization, and volume retention, while reducing necrosis/apoptosis rates (69, 71, 80). Building on this work, Kolle et al. conducted a randomized control trial to assess lipoaspirate-enrichment with ADSCs concentrations up to 2000 times above physiological levels (72, 81). ADSC-enriched groups demonstrated higher volumes of graft retention on MRI at Day 121 (30, 72), and were associated with reduced apoptosis (72, 73). Overall, these findings further suggested that addition of ADSCs may improve graft take by enhancing adipogenesis, supporting angiogenesis and reducing cellular apoptosis (53, 54, 72, 82).

## ADSC Characteristics and Immuno-Profile

Adult stem cells are uniquely able to differentiate into more specialized cell types, replenishing damaged cells to maintain tissue integrity and cellular homeostasis during growth or wound healing (73, 81). Such properties make mesenchymal stem cells (MSCs) prime candidates for use in tissue regeneration (23, 60, 74, 83–86). The clinical use of autologous MSCs for tissue regeneration confers several advantages – chiefly, the ability to avoid host-immune responses. The benefits of ADSCs, are that the yield of stem cells from adipose tissue exceeds that from bone marrow by about 500-fold (75) [5 × 10^5^ ADSCs may be isolated from 400 to 600 mg of adipose tissue (32, 65)], along with superior ease of harvest and minimal donor site morbidity.

Similar to bone marrow derived stem cells (BMSCs), ADSCs are capable of differentiating into a diverse variety of mature tissues (32, 42, 83) – skin, fat, cartilage, bone, muscle, endothelial, and neurogenic cells when cultured with specific induction factors (28, 39, 51, 76, 87).

Apart from this versatile trans-differentiation potential, ADSCs also exhibit an extensive secretory profile consisting of pro- and anti-inflammatory cytokines, chemokines, and growth factors (73, 77–79, 88–91). Whereas, it was previously thought that ADSCs themselves differentiated to replace injured cells [“host replacement” or “building block” repair theories (30, 80, 81, 92, 93)]; secreted paracrine mediators are now thought to perform key active roles in ameliorating RTX and other injuries (54) by orchestrating autocrine or trophic paracrine effects on surrounding tissues (73). The unique secretory profile of ADSCs indicates that they specifically influence the molecular and biological pathways of tissue regeneration (67, 81–83, 94–96), angiogenesis (84, 85, 97, 98), and lymphangiogenesis (20, 86); while suppressing local immune/inflammatory responses (32, 36, 75, 90, 99) and reducing fibrogenesis (39, 100) (Table 1).

**Table 1:** **The postulated regenerative mechanisms of ADSCs in clinical and pre-clinical models of tissue injury**.

Proposed ADSC regenerative mechanism	Experimental findings supporting regenerative mechanism
(1) ADSC adipogenic differentiation	Clinical studies demonstrate newly formed adipose tissue at the site of fat injection resulting in restoration of tissue contour or volume via either (36, 51, 99)
	(a) Direct differentiation of injected ADSC to adipocytes (28, 76); or
	(b) Paracrine stimulation by injected ADSCs, to influence local stem-cell populations to differentiate into adipocytes (44, 92, 101, 102).
(2) ADSC injection increases perfusion of injured tissues through:	(a) Fat grafted sites in murine models of ischemic injury demonstrate GFP or DiI-labeled-ADSCs differentiating to CD31+ endothelial cells *in vivo* (103, 104)
(i) Induction of angiogenesis (ii) Supporting existing vascular structures (iii) Paracrine promotion of angiogenesis	(b) Increased blood vessel density and co-localization of fluorescently labeled ADSC within/near capillaries (95, 103, 105)
	(c) ADSCs form capillary networks on Matrigel matrix and stain positive for vWF (87, 106)
	(d) Release of angiogenic factors by ADSCs promotes re-vascularization and wound healing including: VEGF-A, VEGF-C, VEGF-D, IGF, PDGF-bb, FGF, TGFβ, HGF, IL-6, IL-8, MMP inhibitor 1 precursor, MCP-1, ANG, and SDF-1 (66, 77, 78, 83, 85, 107–110)
(3) ADSCs exert an anti-oxidant effect	(a) Anti-oxidant action provides protection against hypoxia, ischemia reperfusion, and ROS induced damage (81, 111, 112)
	(b) Factors such as hepatocyte growth factor (HGF), G-CSF, GM-CSF, IGFBPs, IL-12, platelet derived growth factor (PDGF-AA), and Superoxide dismutase may mediate these effects (76)
(4) ADSC modulate immune responses, inflammation, and improve wound healing	(a) BMSCs and ADSCs suppress T- and B-cell proliferation via NFkB-mediated mechanisms (32, 66, 113)
	(b) Cytokine and adipokine secretion of IL-6 and IL-8 act as chemo-attractants for monocytes and macrophages, with recruitment to site of injury and promotion of wound healing processes (83, 114–116)
(5) ADSCs modulate granulation tissue, fibrosis, ECM remodeling, and improve epithelialization and wound healing	(a) Alteration of collagen type I and III production by fibroblasts co-cultured with ADSC conditioned media, mediated by down-regulation of genes such as Col3a1
	(b) Up-regulation of type I procollagen a1 mRNA (100, 117)
	(c) Effective migration of keratinocyte and fibroblasts treated with ADSC conditioned media leading to improved re-epithelialization (96, 117–126)
(6) ADSCs secrete lymphangiogenic factors, improving or reversing lymphedema in damaged tissues	(a) Lymphatic fluid stasis results in increased TGFβ1, exerting a further anti-lymphangiogenic effect. Blockade of TGFβ1 along with VEGF-C ADSC stimulation resulted in elevated ADSC expression of lymphangiogenic factors; VEGF-C, lymphatic endothelial cell markers; podoplanin and Prox-1 and increased ADSC survival *in vitro* (86, 127)
	(b) Baseline ADSC production of IL-8, IGF-1, VEGF-D all promote lymphangiogenesis (77, 128)
(7) ADSCs mediate recruitment of endogenous stem cells via a homing chemokine gradient	(a) Murine models have MSC homing to site of injury. Systemic injected human MSCs migrated and engrafted at the site of ischemic or necrotic injury (44, 48, 123–126, 129)
	(b) Stromal derived factor 1α (SDF-1α) secreted by ADSCs is the main chemo-attractant of systemic stem cells to the area of injury (112, 130)

Since their initial description, the cell surface molecular marker profile of ADSCs has remained controversial (51, 131), predominantly due to differences between post-extraction purification protocols, culture conditions, and variations in the use of whole or sub-total SVF (36, 40, 62, 88–90). The International Society for Cellular Therapy defines ADSCs as cells that demonstrate plastic adherence in standard tissue culture conditions (91, 132), express a surface marker profile of CD34^+^, CD31^−^, and CD45^−^ (2, 5, 20, 21, 67, 92–95, 98, 133–135) (Figure 2) and demonstrate multi-potent “tri-lineage” differentiation capabilities – i.e., differentiation into bone, cartilage, and fat (101).

## Oncological Safety of ADSCs in RTX-Treated Cancer Beds

Questions regarding oncological safety of fat grafting following cancer clearance have been raised (36, 129, 136). While long-term tissue changes following fat grafting may impede radiological surveillance for cancer recurrence (62, 137), Delay et al. state that experienced breast radiologists should be able to differentiate “post-graft” from malignant calcifications (99). The major oncological concerns relate to the beneficial properties of ADSCs in RTX-injury potentially also promoting tumor growth in areas previously treated for cancer (59, 60, 132–134, 138–140). Molecular adaptations that promote graft survival include secreting hypoxia-induced growth factors such as VEGF-A or VEGF-D (see below) – both of which induce angiogenesis and lymphangiogenesis (107, 108, 113, 135, 141) – stimulating breast cancer growth and metastases (26, 59, 60, 129, 130, 132–140, 142–146). Krumboeck et al. found that while ADSCs may not necessarily trigger transformation of quiescent tumor cells to active growth, they could promote proliferation of residual cells after cancer resection and/or adjuvant therapy (60, 147). In contrast, proponents of fat grafting argue that *in vitro* models may not be representative of human tumors (144, 145). In light of evidence to date, Claro et al. and Zimmerlan et al. call for postponement of “stem-cell enhanced” fat grafting for breast reconstruction until long-term follow-up data becomes available (42, 44, 60, 132, 145, 148). Gutowski et al. proposed screening to exclude high-risk patients (e.g., with BRACA1/2 mutations) from fat grafting (51, 146). Nevertheless, fat grafting for breast reconstruction has been reported in over 3,000 patients in published studies (147, 149). While systematic reviews of current practice examined clinical efficacy, the lack of randomized controlled trials examining oncologic safety and insufficient follow-up of smaller studies mean that no clear conclusions have been reached (32, 54, 96, 108, 145, 148). Overall, a more detailed understanding of mechanisms by which fat graft may reverse RTX-injury – and how these pathways may cross-talk with the regulation of tumor growth are required.

## ADSCs and Radiotherapy-Induced Soft-Tissue Injury

Adipose-derived stem cell in the setting of RTX soft-tissue injury raises two broad questions:

(a) The effects of injury on ADSCs.
(b) How ADSCs specifically modulate RTX-Injury.

### Effects of injury on ADSCs

#### Radiotherapy injury, adipocytes, and the SVF

Injury induced by RTX has previously been explained by rapid, extensive necrotic, or apoptotic cell death in the stem-cell and progenitor populations (23). However, as neither of these mechanisms fully account for the chronic, progressive, and evolving nature of RTX-injury in soft-tissues (10, 24), “sub-lethal” changes such as premature senescence, terminal differentiation, or reproductive cell death have been implicated (23, 48, 150). More recent findings suggest that ADSCs display radio-resistance compared with other components of SVF such as adipocytes (150). This may be explained by a greater ability of MSCs to retain their proliferative capacity due to superior DNA damage repair mechanisms compared with those found in terminally differentiated cells (150). Bill et al. suggested that terminal differentiation of cells may correlate with increased G1-cell cycle arrest and reduced ability to repair RTX-induced double-stranded DNA breaks (151). Additionally, reduced metabolic demands of steady-state ADSCs may protect them from hypoxia and subsequent apoptosis, enabling their preservation in order to perform regenerative functions (39, 152).

As ADSCs share many regenerative properties with BMSCs, much of our understanding of mechanisms by which healthy ADSCs modulate RTX-injury has been extrapolated from BMSC studies (54, 153). Ponomaryov et al. demonstrated that sub-lethal RTX-injury to BMSCs resulted in an increased expression levels of SDF-1 (also the main chemotactic factor for ADSCs) at both mRNA and protein level (130). This increased SDF-1 expression in-turn mediated homing of CXCR4^+^ uninjured stem cells via a chemokine gradient (130). This gradient is integral to homing and importing uninjured ADSCs, as surviving ADSCs originating within the injured area may be significantly functionally impaired (32, 77, 86, 96, 106, 108, 114, 118, 119, 127, 154). Poglio et al. characterized the effects of RTX on murine adipose tissue primarily as decreasing adipocyte size and number, increasing ROS, and impairing SVF proliferation and adipogenic differentiation (25). While the overall composition of the SVF was unaltered by irradiation, the authors concluded that changes to the capacity of cells within the SVF to proliferate or differentiate could impair the regenerative properties of fat graft (25), as demonstrated by Li et al. in irradiated BMSCs, which displayed suppressed proliferation, osteogenesis, and adipogenesis (155). A further mechanism of action of ADSCs maybe a similar recruitment of and differentiation toward a fibroblastic phenotype seen in irradiated BMSCs (32, 156).

Functional cellular analysis performed by Schonmyer et al. suggested that irradiated murine BMSCs underwent low-level spontaneous osteoblastic differentiation, in preference to adipogenic or chondrogenic lineages (156). Furthermore, attenuation of the response of irradiated BMSCs to stimulation with lineage-specific differentiation media was decreased in irradiated cells and was associated with down-regulation of bone-specific markers (ALP and osteocalcin) and adipose-specific markers (lipoprotein lipase, C/EBPb, and leptin) (156). These findings further highlight the altered capacity of stem cells to respond to cues in their microenvironment to replenish damaged cells, following RTX (156). Mechanistically, alterations to paracrine signaling via Wnt10b and Sirtuin-1 (a subset of a family of proteins that regulate stem-cell differentiation) were also seen to mediate altered adipogenesis and osteogenic differentiation characteristics in BMSC (157). Meanwhile, another subset of the same protein family, Wnt3a and Wnt5a, were found to be up-regulated in RTX-injury and may additionally induce senescence in irradiated BMSCs (30).

### How ADSCs specifically modulate RTX-injury?

The original “building block” theory that stem cells migrate to an area of injury to differentiate and replace the injured cell has been superseded, as only a small number of grafted cells – of which ADSCs make up an even smaller proportion (44, 154) – survive the fat transplant injection (42, 77). More recently, paracrine mechanisms such as immune-modulation and the generation of protein growth factors secreted by surviving grafted ADSCs, have gained favor (51, 54, 102, 106, 114, 149, 158). Walter et al. demonstrated modulation of keratinocyte and fibroblast migration in response to BMSC conditioned media, in which analysis of the paracrine secretory profile detected increased expression of IL-6, IL-8, MCP-1, and to a lesser degree RANTES and TGFβ1 proteins (76, 119). The key differences in the protein growth factor profiles of the two types of MSCs as shown on cytokine array studies were IL-8, IGF-1, and VEGF-D, which were secreted by ADSCs but not BMSCs (77). Given that the mechanisms underlying the overall profile of RTX-injury appear to involve poor vascularity, hypoxia, and lymphedema – and that these three growth factors are implicated in each – it seems intuitive that ADSCs play a critical role in reversing these micro-environmental changes. This protein secretion profile indicates that ADSCs may facilitate angiogenesis and lymphangiogenesis, in addition to the simple anti-fibrotic effects with which they have been previously associated with (36, 100, 113, 141). However, further detailed systematic analysis of the secretory expression profiles of ADSCs is required to identify which specific growth factors are released, under which conditions, and how they may modulate the wound healing, angiogenesis, and lymphangiogenesis (81, 103). Such an effect was typified by the down-regulation in VEGF-A production by ADSCs in response to irradiation, as shown by Ebrahimian et al. (87).

## ADSCs and Angiogenesis in Hypoxia

Radiotherapy, particularly associated with subsequent surgery, creates tissue hypoxia by up-regulating expression of inducible transcription factor HIF-1α, either through generating ROS, Nitric Oxide, or inducing macrophage recruitment or release of stress granules (159). *In vitro*, the constituent components of adipose tissue each responded differently to hypoxic stress stimuli in a study by Haubner et al. (44). These authors found that adipocytes, and to a lesser degree, endothelial cells, underwent apoptosis in hypoxic conditions, while ADSCs displayed superior cell viability (44); a finding verified by Frazier et al. in a viability study (160). Other authors further suggested that the superior survival capacity of ADSCs facilitates their contribution to active repair of adipose tissue (44, 48, 85, 161), and that stem cells are maintained in a baseline state of relative hypoxia, enabling them to derive protection from cyto- or genotoxic stressor by utilizing anaerobic metabolism (106, 159). Alternative hypoxic pre-conditioning models such as mechanical thermal stress or nutrient deprivation have also shown superior stem cells survival, in addition to a modified paracrine secretory profile (35, 81, 84, 85, 106, 107, 154, 160, 161). Unsurprisingly, much of this hypoxia-induced growth factor expression profile is pro-angiogenic. Examples include HIF-1α and SDF-1a production (84, 160), which in turn increased secretion of pro-angiogenic and anti-apoptotic cytokines VEGF-A, hepatocyte growth factor (HGF), bFGF, by up to fivefold in spheroid models (26, 81, 107, 111, 161, 162). Frazier et al. found that ADSC-conditioned media (ADSC-CM) from cells grown in hypoxic conditions demonstrated altered protein levels of Fibronectin 1, TGFβ1-induced protein, Osteonectin, and Collagens (Type 1a1 and 1a2), potentially also facilitating angiogenic sprouts through the ECM (111, 160). Despite this compelling pre-clinical work, increased proliferation, migration, or sprouting may not necessarily correlate with the formation of functional vasculature or enhanced tissue perfusion *in vivo*, without the vessels first acquiring adequate vessel stability (109, 162). A study investigating the role of ADSCs in stabilizing endothelial networks attributed them with properties akin to those of pericytes, which act synergistically with blood endothelial cells (BECs) to contribute to neo-angiogenesis. These ADSCs were specifically shown to establish neo-vessel connections with the pre-existing local vasculature and conducted blood flow as a stable network (163). In addition, hypoxia and ischemia have been independently observed to induce trans-differentiation of ADSCs into CD31^+^/VWF^+^ BECs that may also contribute to the establishment of neo-vasculature (96, 103, 104, 109). Overall, ADSCs may contribute to angiogenesis by promoting paracrine effects that stabilize neo-vasculature, by supporting existing RTX-damaged blood vessels, or finally, by differentiation into BECs that integrate into forming vessels (103, 164). Local or systemic injection of labeled-ADSCs following body wall RTX treatment were associated with increased angiogenesis consisting of perivascular aggregation of CD31^+^ ADSCs, which was interpreted as trans-differentiation of ADSCs to BECs (87, 112, 164).

In addition to pro-angiogenic effects, ADSCs were also shown to display protective effects on non-vascular cells in hypoxic conditions. Lee et al. demonstrated anti-apoptotic effects in dermal fibroblasts, which developed enhanced resistance to oxidative stress when treated with ADSC-CM (76, 111). Similarly, anti-oxidants superoxide dismutase and glutathione activity was enhanced in cell cycle analyses of fibroblasts cultured in ADSC-CM (111). In a pre-clinical model of ischemia reperfusion injury, Uysal et al. injected ADSCs into axial flaps, subsequently clamping then finally unclamping the vascular pedicle to allow reperfusion. They showed enhanced flap viability and up-regulated expression of VEGF-A, TGFβ, and FGF proteins detected immuno-histochemically (109). Collectively, these findings suggest that ADSCs produce growth factors that may ameliorate ischemic insults and can exert a protective effect against reperfusion injury (76, 109).

## Mechanisms of ADSC-Mediated Reversal of Radiotherapy-Induced Soft-Tissue Injury

**Figure 3:**
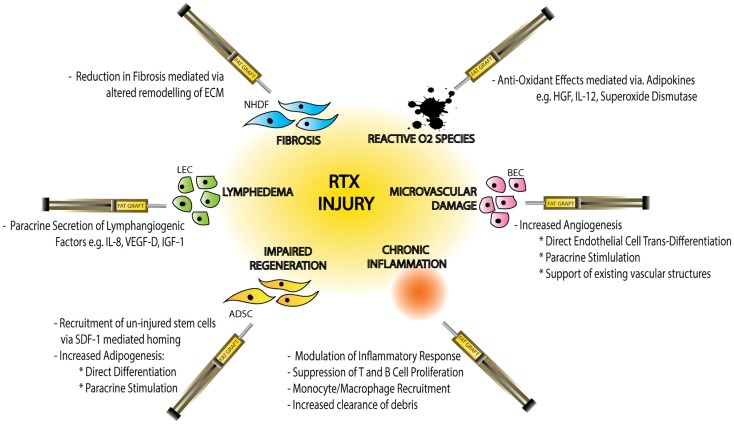
**Schematic diagram demonstrating the effects of radiotherapy (RTX)-injury on individual cellular components, the resulting clinical manifestations of injury and the mechanisms by which fat graft may ameliorate this soft-tissue injury**. Normal Human Dermal Fibroblasts (NHDF), extracellular matrix (ECM), hepatocyte growth factor (HGF), interleukin-12 (IL-12), blood endothelial cell (BEC), adipose-derived stem cell (ADSC), stromal derived factor-1 (SDF-1), lymphatic endothelial cell (LEC), interleukin-8 (IL-8), vascular derived growth factor-D (VEGF-D), and insulin-like growth factor-1 (IGF-1).

In addition to anti-hypoxic effects ADSCs have also been shown to mediate alternative paracrine responses to RTX-injury including anti-inflammatory and anti-apoptotic effects (Figure 3).

In an investigation of the effects of irradiation on BECs, Haubner et al. demonstrated up-regulated expression of inflammatory cytokines IL-6, FGF, ICAM1, and VCAM1. Co-culture with ADSCs in this model demonstrated reversed expression of all the detected inflammatory cytokines (66). Similarly, Chang et al. used a model of intra-peritoneal ADSC injection following abdominal irradiation to demonstrate a significant reduction in inflammation in ADSC-treated animals, with enhanced intestinal re-epithelialization and improved survival rates. ADSC injection was associated with increased serum levels IL10, VEGF-A, bFGF, and EGF as well as enhanced SDF-1-mediated recruitment of hematopoetic stem cells to the site of injury (112). Also in the upper gastrointestinal tract, Lim et al. and Kojima et al. demonstrated protective and anti-apoptotic effects of ADSC injection in a model of RTX-induced salivary gland injury (165, 166).

Finally, the dermal and subcutaneous responses to ADSC injection in animal models of both in chronic RTX-wound healing and intact irradiated skin, manifested as increased dermal thickness quantified by a reductions in fibrotic marker Smad-3 and a collagen-based scar index measurement (164, 167). An equivalent large animal model of ADSC-enriched fat graft injections following localized RTX demonstrated integration of q-dot-labeled-ADSCs into the dermis, with associated favorable wound healing, enhanced epithelialization, increased subcutaneous adipose tissue, and reduced apoptosis; along with recruitment and activation of lymphoid cells (83, 168).

## Future Directions and Conclusion

Significant improvements in cancer therapy have lead to improved cancer survival, meaning that more patients are living longer with the after-effects of RTX. The resulting fibrosis, lymphedema, and impaired tissue quality characteristically reduce the patient’s quality of life and complicate subsequent surgery. Recently, fat grafting has been added to plastic surgeons’ armamentarium to combat RTX-induced soft-tissue injury. Studies demonstrate the multifaceted nature of ADSC-driven tissue regeneration via enhanced angiogenesis and adipogenesis, while also mediating anti-apoptotic, anti-fibrotic, anti-oxidant, and immune-modulatory properties.

Authors who investigated the effects of injurious stimuli such as hypoxia and radiotherapy on ADSCs have demonstrated a superior ADSC survival capacity compared to other cellular components of fat grafts, through utilization of anaerobic metabolism. However, the sub-lethal RTX-induced injuries impair ADSC proliferative capacity, responsiveness to environmental differentiation cues and alter the ADSC paracrine secretory profile. Such functional alterations in injured ADSCs may account for the inability of local ADSCs to replenish surrounding tissue following radiotherapy injury, thus necessitating the introduction of un-irradiated fat (and ADSCs) in the form of a fat graft. These functional ADSCs may reverse radiation injury by restoring the normal proliferative and differentiation capacity of the local ADSC population.

In conclusion, while *in vitro* and *in vivo* models demonstrate the benefits of fat grafting, more comprehensive cellular and molecular analyses using genome-screening platforms are needed to elucidate the true mechanism behind ADSC-mediated reversal of RTX-injury. A detailed understanding of the reaction of individual cell types in response to RTX-injury is required in order to treat pathological processes such as fibrosis, lymphedema, and hypoxia – which contribute to the formation of RTX-induced soft-tissue injury. ADSCs may possess these characteristics; however, a targeted molecular therapy that harnesses the beneficial effects of ADSCs, without raising the potential of enhanced tumor growth, activation, or metastases is required.

## Conflict of Interest Statement

The authors declare that the research was conducted in the absence of any commercial or financial relationships that could be construed as a potential conflict of interest.
